# Substitution and Its Attendant Evils

**Published:** 1892-09

**Authors:** John Aulde

**Affiliations:** Philadelphia, Pa.


### SUBSTITUTION AND ITS ATTENDANT EVILS.

BY JOHN AULDE, M. D., PHILADELPHIA, PA.

The evils attendant upon substitution and sophistication of remedial agents, have long been surmised; they have not, however, until recently, received attention at the hands of the medical profession. Increased diagnostic skill, along with greatly improved facilities for the manufacture of medicaments, favor an approach toward mathematical exactness in computing therapeutic results. When these are wanting, we challenge the character of the remedy. The question which presents itself is: Has our patient received the true medicament, or a base counterfeit? However attractive in theory, it will be found impracticable for the medical profession to drift away from the pharmacists, and it should be our aim to reward the faithful and bring the guilty to punishment. The friendly bond between the two professions should be honesty, as neither can afford to work independently; there is an interdependence which makes them mutually helpful.

It is said of Lawson Tait, that he has returned to first principles, and carries a mill with him, so that when ergot is needed, he prepares it fresh with his own hand. The reliable character of Squibb's ether has been maintained through his business sagacity, in having it prepared chemically pure and distributed all over the world in sealed cans, thus precluding the possibility of sophistication or substitution.

The life of a patient suffering from rheumatism may depend upon his being supplied with sodium salicylate prepared by a combination of Merck's chemically pure bicarbonate of soda, and true salicylic acid obtained from oil of wintergreen, and yet, few pharmacists even in large cities, pretend to keep either in stock. They are the exception in Philadelphia, and doubtless the same is true of other cities.

Some years ago, Dr. Squibb, of Brooklyn, set his seal on Marchand's peroxide of hydrogen, by endorsing its character and defending its merits as the most powerful and yet harmless bactericide which could be employed in the treatment of various formidable and fatal diseases. Dr. Robert T. Morris, Dr. Paul Gibier and other well known authorities have corroborated his statements from clinical observation, and as a consequence, a revolution has taken place in our methods of treatment in both medical and surgical practice. The efficacy of this simple remedy, its innocuousness and extended field of application, have shed a flood of light upon modern therapeutics, but at the same time, there has followed in its train, a host of worthless imitations.

The substitution of the commercial for the medicinal peroxide, is calculated to work serious injury, and destroy our confidence in a most potent remedy. In the treatment of diphtheria, for instance, the commercial product is positively harmful. When death results, shall we blame the attending physician or the unscrupulous druggist who substitutes a base imitation for the genuine product? And still, pharmacists who claim to be respectable, do not hesitate to trifle thus with human life. Is it any wonder then, that our mortality percentages are on the wrong side?

Cascara sagrada has been counterfeited and sophisticated, until it is almost impossible to secure a reliable preparation of this most useful medicament, although Parke, Davis & Co., the pioneers in its introduction, have adopted every means in their power for the protection of the medical profession.

Antipyrin, a patented preparation, has met with phenomenal sales, and possesses distinct therapeutic properties, and as a result, imitations and substitutes are offered to take its place in medical practice. Whether these imitations are better or worse than the original product, I do not care to discuss; neither is it for the druggist to decide. The decision here, as to any special remedy or preparation, rests entirely with the physician, as he alone is responsible for the condition of the patient; no one else, not even the druggist, should be permitted to interfere with his directions. Substitution is an evil which should be guarded against; it is an evil which must be eradicated or the entire medical structure will collapse. It is a duty we owe to ourselves and to our patients, to look after this unnatural condition of affairs in which we are so vitally interested, and the time is near at hand when a systematic effort must be made, with a view to accomplish the desired end.

This subject is commended to the attention of the American Medical Association, with the suggestion that a committee be appointed who who shall recommend suitable measures for the protection of the medical profession against the evils of substitution and sophistication on the part of unscrupulous pharmacists. Shall we have a "list"?—*Journal of the American Medical Association.*

